# Maggot Debridement: An Alternative Method for Debridement

**Published:** 2011-07-12

**Authors:** Finn Gottrup, Bo Jørgensen

**Affiliations:** Copenhagen Wound Healing Center, Department of Dermatology, Bispebjerg University Hospital, Copenhagen, Denmark

## Abstract

Debridement is an essential component to promote healing in a problem wound. Several techniques are available including maggot debridement therapy (MDT). **Objective:** To describe the efficacy of MDT for treating problem wound especially diabetic foot ulcers. **Methods:** The topic is elucidated from different points of view: the mode of action, when to use, use in a practice, clinical results, and discussing the problem of creating evidence for the clinical effect. **Results:** Literature and own results demonstrate that MDT is a safe method with few side effects. Maggot debridement therapy is as good as or better than conventional often surgical debridement, is more selective than surgical debridement, decreases time to healing and stay of patients in the ward, and may decrease the risk of major amputations. However, the evidence of these effects of MDT on the highest level is presently lacking. A detailed description of how to use MDT in practice is provided including a visual demonstration in a video. **Conclusion:** In spite of lacking clinical evidence, MDT clinical experience strongly suggests that this technique is effective and safe. It can be used for most types of problem wounds, but our indication is primarily diabetic foot ulcers, because of its selectivity for debriding necrotic dead tissue. It may be a valuable alternative surgical/sharp debridement.

Previously, necrotic tissue like eschar or scab often like pus was looked upon as a natural part of the healing process.^1^ More recently, however, it has been known that tissue necrosis influences the healing process in a negative way resulting in a delayed or even stopped healing process. Wound debridement is for these reasons a vital part of the treatment and healing of problem wounds.

*Wound debridement* is defined as the removal of foreign material and dead contaminated tissue from (or adjacent to) a traumatic or infected lesion to expose healthy tissue. It may also include removal of foreign material that has become embedded in the wound.^2^

The main reason for debriding a wound is to avoid substratum for bacterial growth,^3^,^4^ ongoing inflammation, and leukocyte infiltration with delayed progression to the proliferative and remodelling phases of wound healing,^5^,^6^ compromised restoration of the structure and function of the skin,^7^ odor management issues, and other negative effects.

Debridement is perhaps the most important part of the concept “Wound Bed Preparation” and wound management in general. Debridement must, however, not be seen in isolation but should be regarded as one element to achieve healing.^8^

Several types of debridement are available.^9^,^10^ The most generally known is surgical or sharp debridement, but nonsurgical methods have increasingly achieved attention during the last years: enzymatic agents, chemical agents, autolytic debridement, biosurgical debridement, wet-to-dry dressings, whirlpool and hydrotherapy, high-pressure irrigations, and some other types of debridement. Debridement has been subdivided into selective and nonselective methods depending on whether only nonviable tissue is removed. Selective debridement methods can, for example, be mentioned as partial surgery, use of enzymes, osmotic agents, autolysis, and maggot therapy.

The final choice of debridement method is based on the wish of achieving the most rapid, safe, and painless healing of the wound. To accomplish this goal, the debridement has to be sufficient. This is attained when the wound bed consists entirely of healthy tissue.^12^

The beneficial effects of maggots in the process of wound healing are known for centuries. Since the last 15 years, maggot debridement therapy (MDT) is used in clinical practices in Europe and the United States for the treatment of various types of severely infected and necrotic wounds with successful healing results.^13^,^14^ Several historical documents prove that in ancient times, maggots, also known as “biosurgeons,” were already applied for wound treatment.^15^ The Aboriginals in Australia and Maya tribes in Central America used larvae frequently to clean wounds. William Baer (1872-1931), orthopedic surgeon at the John Hopkins Hospital in Baltimore was the first surgeon that employed larvae of the Lucilia sericata type for the treatment of children with osteomyelitis in 1929.^16^ Baer described a fast debridement, the reduction of bacterial amounts, a decreased odor and alkalinization of the wound surface. Until the 1940s American surgeons used MDT, but the discovery of Penicillin by Alexander Fleming in 1928 and the widespread production and use of this first antibiotic from 1944 leaded to the disappearance of maggots as a treatment for infected wounds.^17^ However, only 4 years after the introduction of Penicillin, more than 50% of all *Staphylococcus aureus* specimens produced β-lactamase, which made them resistant to the mould.^17^ Bacterial resistance to Penicillin, and also to other types of antibiotics, increased in the time afterwards, which resulted in the failed healing of infected wounds, and because of this, maggots made their comeback in the late 1980s.^18^ In the following years, MDT was reintroduced in United States and Europe using maggots of the type Lucilia sericata.^19^^-^22

The aim of this clinical article is to describe the mode of action, when to use MDT, the practical use of MDT in debridement, clinical results, and discuss the problem of creating evidence for the clinical effect of MDT.

## METHODS

### Mode of action of MDT

Maggot's debridement has been suggested to work by mechanical and biochemical techniques.^23^,^24^ Mechanical debridement is caused by the specific mandibles or “mouth hooks” of the maggots and their rough body which both scratch the necrotic tissue. Furthermore, maggots produce excretions and secretion (ES) that possess proteolytic enzymes that can dissolve the dead and/or infected matrix on the wound bed.^24^ However, the long-standing hypothesis was that the mechanical debridement was one of the responsible mechanisms for the effectiveness of MDT, but the current studies do not support this objective and show more evidence for the biochemical mechanisms underlying to its success.^25^

Maggots excretions and secretions contain allantoïn, sulfhydryl radicals, calcium, cysteine, glutathione, embryonic growth stimulating substance, growth stimulating factors for fibroblasts, carboxypeptidases A and B, leucine aminopeptidase, collagenase, and serine proteases (trypsin-like and chymotrypsin-like enzymes, metalloproteinase and aspartyl proteinase).^26^

A recent in vivo study possibly supports the theory that the direct mechanical action of free-range maggots is limited. In this research, larval therapy with free-range maggots and maggots in Biobags was compared with hydrogel application and showed faster debridement with the maggots.^27^ Although the maggots in Biobags needed 28 days to debride and free-range maggots needed only 14 days, both therapies were very effective in debridement compared to hydrogel, which cleaned the wounds in 72 days.

Maggot ES can prevent, inhibit, and break down biofilms of various bacteria on commonly used prosthetic materials, and thus, it may in the future provide a new treatment of biofilm-associated infections of orthopedic biomaterials.^28^ On the contrary, the antimicrobial effect seems limited against pseudomonas biofilm. An in vitro study has shown reduced antimicrobial effect against Quorum sensing controlled pseudomonas aeruginosa virulence factors.^29^

The type of maggot used in MDT is Lucilia sericata, whose actions are limited to the necrotic wound and which spares the healthy tissue. Table 1 is summarizing the expected mode of action for maggot debridement.^30^

*Side effects*: There are no severe side effects reported on MDT. Sometimes, a tickling feeling of the crawling maggots is noted; however, after using the captured method, there are fewer complaints about this sensation. Exceptional cases, for example, from patients with leg ulcers, who are suffering from ischemic disease, report an increased pain by MDT,^31^ and in spite of neuropathy in diabetic foot ulcers (DFU), a type of pain can be experienced in a few of these patients. The origin of reported pain during maggot application is not known, because the wound-healing effect of maggots is not related to their direct crawling action in the wound surface. No allergic reactions were ever noted.

### When to use MDT

*Indications/contraindications*: Maggot debridement therapy can be used for acute and chronic wounds requiring debridement. In literature, the reported success rate varies from 80% to 90%.^32^ Clinical relevant indications are DFU, ischemic leg ulcers, osteomyelitis, burn wounds, as postoperative treatment for a necrotizing fasciitis or for the prevention of (further) amputations.^19^,^32^^-^34 In venous leg ulcers, however, maggot seems to have limited effect.^27^

Maggot debridement therapy is contraindicated when there are open wounds into the abdominal cavity, because of the risk of organ lesions. Other contraindications are pyoderma gangrenosum in patients with immunosuppressive therapy and septic arthritis.^31^,^35^ Caution is advised in treating wounds near to large arteria and veins. Wounds heavily contaminated with pseudomonas aeruginosa may have limited effect of maggot debridement.^29^ Very dry wound may be a relative contraindication because maggots require a moist environment.^20^

### Practice clinical use of MDT

**Figure 1:**
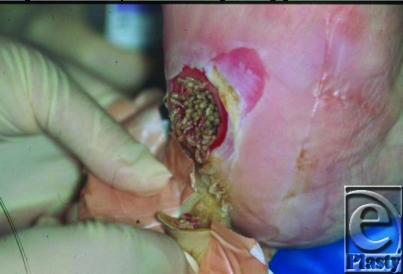
Freely crawling maggots.

Currently there are 2 modes of application of MDT. First freely crawling maggots (Fig 1) can be applied to the wound bed and covered by a nylon net. On the top of this is placed a gauze bandage to keep the maggots captured in the wound and to let them breath freely. A quantity of up to 10 maggots per square centimeter wound surface for 3 days consecutively are used in our institution. After this period, the maggots should be removed by washing out the wound by saline.

**Figure 2:**
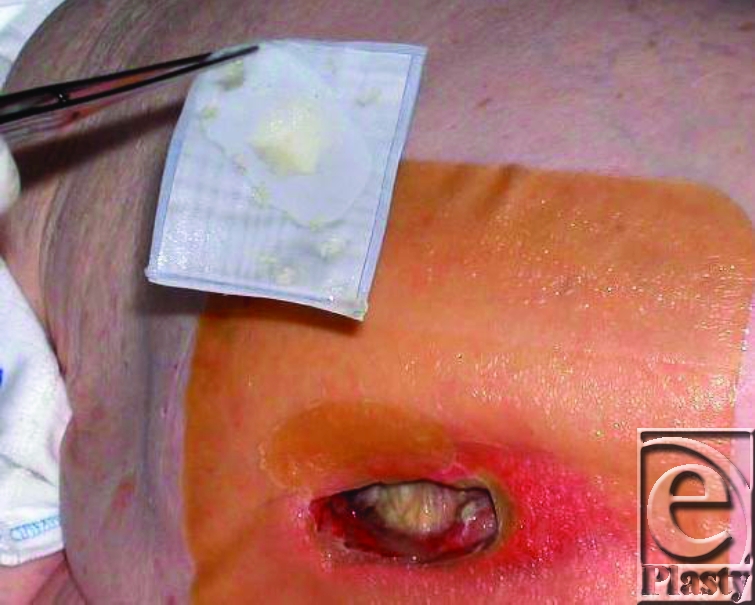
Maggots in a bag.

In the second mode, maggots are captured and enclosed in special biobag containing a polyvinyl alcohol spacer^36^ (Fig 2). The network of the biobag is permeable and permits the migration of maggot ES to the wound. This bag facilitates the application of MDT and also the inspection of the wound bed during the treatment at any time. The effectiveness of the MDT captured in bags or in free-range application seems to be equal,^36^ but in case of complicated undermined cavity wound-free maggots may be preferable. We advise to use a quantity of 5 to 10 maggots per square centimeter wound surface for 3 to 4 days consecutively after which the bags containing maggots should be replaced in combination with a saline cleaning of the wound.^37^ Furthermore, it is necessary to use a physiological saline solution daily to keep the surface wet. It has, however, to be born in mind that excessive flushing could drown the maggots. In addition, it is advised to change the covering gauze bandage daily to prevent odor and avoid the dressing to be filled with wound fluid, which could drown the maggots.

Sometimes, there are concerns about the resistance from patients to the utilization of MDT, but many reports show in accordance with our observations that patient acceptability is high.^38^ Information materials in form of handouts and posters is facilitating acceptance in patients and relatives.

## RESULTS

### Literature

Maggot debridement has been used on different types of problem wounds like pressure ulcers,^39^,^40^ venous leg ulcers,^27^,^41^ DFU,^42^^-^45 peripheral arterial diseases,^46^ and acute surgical wounds as treatment^47^ and as preparation for surgical wound closure.^48^

Maggot debridement therapy has been used for both out- and inpatients and reported to have a success rate between 67% and 88%.^31^,^39^,^44^,^49^^-^51 Nevertheless, studies with direct comparison between a maggot and control group has only been lesser performed. In a study on pressure ulcers, MDT showed a success rate of 80% compared to 48% when using conventional debridement.^40^

Diabetic foot ulcer patients are a major indication for MDT, and some comparative studies have been published. In a study on DFU,^44^ conventional therapy after 5 weeks showed that in this group more than 33% of the wound surface still was covered by necrotic tissue, while all wounds in the MDT group after 4 weeks were completely debrided. Both studies showed a shorter stay on the ward of the hospital, and one study has demonstrated a lower need for amputations after MDT.^51^ In patients with neuroischemic diabetic foot wounds and peripheral vascular disease, it has been demonstrated a shorter healing time after MDT, a 3 times higher risk for undergoing amputation in the control group and significant more antibiotic-free days in the MDT group.^46^

Similar results were shown in another comparative DFU study. It was shown that MDT (using Lucilia cuprina maggots) is as effective as conventional surgical debridement, and there was an overall amputation rate of 20% in the MTD group compared with 38% in the surgical debridement group.^44^ The outcome and decrease in amputation level, however, did not reach statistical significant, perhaps because of the low number of patients included in the study.

In a recent randomized controlled trial (RCT) in venous leg ulcers,^27^ 267 patients were included. It was found that healing was not significantly different between the loose or bagged larvae group and the hydrogel group, but the larvae therapy reduced the time to debridement. In this study, mean ulcer-related pain scores were higher for both types of larvae treatment than for hydrogel treatment.

A few studies have focused on the cost-effectiveness of MDT. Wayman et al^52^ prospectively measured personal and material costs of treating venous leg ulcers with MDT or hydrogel. The cost of treatment per patient in the MDT group was £79 compared to £136 for the hydrogel group (*P* < .05). The MDT group also required fewer visits to achieve full debridement than controls. If nursing costs were included the total expenditure to full debridement of one wound, the difference would have been 6 times lower in the MDT group.^53^ For DFU, it has been calculated that the use of MDT may save almost £50 million annually in the United Kingdom.^54^

### Practice in Copenhagen Wound Healing Center (CWHC)

Practice in Copenhagen Wound Healing Center is a specialized wound healing institution established as a full-integrated hospital unit in the socialized government health care system of Denmark.^55^^-^58 The Center consists of outpatient clinics and inpatient wards with 15 beds only for patients with severe wounds of all etiologies. The multidisciplinary staffs consist of doctors (surgeons, dermatologists), nurses (specialized), podiatrists, physiotherapists, researchers, etc.

In DFU patients, especially debridement, treatment of infection, wound phase—specific conventional local wound treatment, offloading, and arterial revisualization have been implemented. Implementation of this treatment has reduced the major amputation rate to 20% of what it was 15 years ago. All types of modern debridement systems are used including MDT.

The CWHC introduced MDT in Denmark and presently more than 300 patients have been treated and the method to treat has been locally published.^59^,^60^ The main indication has been DFU. The reason for choosing this indication is that it is critical in this group of patient to provide a type of debridement, which is as selective as possible to avoid tissue damage as much as possible. In the DFU patient, exposed bone, especially at the heel area, often results in a major amputation. The maggots are in this case much more selective than the surgeon performing a sharp debridement. In CWHC, all DFU treatments have been performed as an inpatient procedure.

The first 16 DFU patients treated by MDT in CWHC were presented in 2002 in Dublin and Oxford. The wounds were of significant size (mean = 31 × 47 mm), and all wounds were necrotic. Although 44% of the patients had critical ischemia (toe pressure < 30 mm Hg), a total of 88% had an improvement of granulation tissue development after MDT. Total healing was achieved in 25% patients, whereas 19% of the patients, all with a toe pressure of less than 30 mm Hg underwent major amputation.

Since then, we have been using MDT routinely as a debridement method in case of severe necrosis except for dry black necrosis, which need to be removed before the use of maggots. This has resulted in the recommendations for the procedure of MDT shown in Table 2.

## DISCUSSION

This clinical article describes the mode of action, the time when to use MDT, the practical use of MDT in debridement, clinical results, and practical recommendations. Maggot debridement therapy has been shown as a safe and cost-effective treatment in contrast to the less selective surgical debridement and the use of expensive antibiotics. It has further been shown that patients treated for MDT have a shorter stay at the hospital ward and patients treated for DFU have a reduced need for amputation, and in these types of patients an improved or at least a comparable effect to conventional debridement in terms of outcome have been found.

Why is MDT not yet a fully integrated debridement technique worldwide? This is probably related to the sparse evidence on the highest level available for the effect of MDT. Most of the studies on debridement^61^,^62^ and maggots are relatively small and present insufficient numbers to fulfill the Cochrane system demands, and the conclusion then is that there is insufficient evidence of the effects and more research is needed.

Similar problems are found for wound management in general, where a paucity of high-quality evidence is found, because studies often are based on inadequate sample size, short follow-up, nonrandom allocation to treatment arms, nonblinded assessment of outcomes, poor description of control, and concurrent intervention.^63^,^64^ The ongoing dilemma is to address the requirements of a high-quality RCT, as demanded by regulatory authorities, and to produce evidence relevant to clinicians working within the field.^65^ Another major issue relates to the choice of an appropriate population and endpoints to test the value of a specific intervention in a specific condition.^66^ A successful and accepted outcome for high-quality RCT, therefore, is linked to an adequate sample size (number of patients), sufficient follow-up time, random allocation, blinded assessment of outcomes, correct description of control, and concurrent intervention. These issues will be very difficult to fulfill in a RCT where MDT is one of the investigated arms but should be taken into account every time a study protocol is completed.

Besides the structure of the protocol and clinical effect of MDT, future studies should try to further clarify the mechanism behind MDT. An important area to investigate is the composition of maggot ES and determine the effective substances that are responsible for the debridement, including the remodeling effect on the extracellular matrix components and the reduction of biofilm formation. In a recent defended PhD thesis, 38 different proteases and new insect defensin (a type of antibiotic) were detected.^67^

## CONCLUSIONS

The maggots of Lucilia sericata are especially indicated for wounds that need debridement. At present, 2 modes of application are available—the free range and the captured method. Both methods are effective, but in undermined cavity wound-free maggots may be preferred. Maggot debridement therapy also reduces biofilm formation and has a positive effect on extracellular matrix components.

Even though clinical evidence on the highest level of the effect of MDT is lacking, the clinical experience strongly suggests that the technique is an effective and safe method of debridement for some wound patients such as those with DFU.

## Figures and Tables

**Figure 3:**
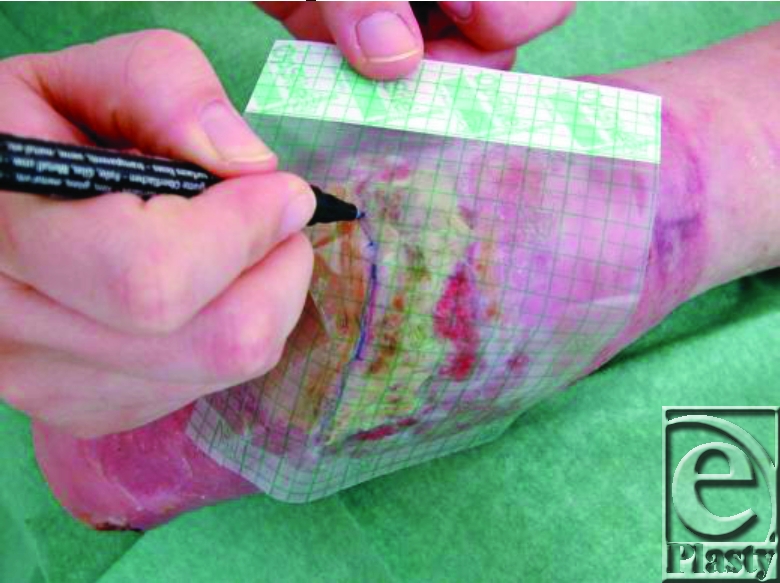
Drawing the wound.

**Figure 4:**
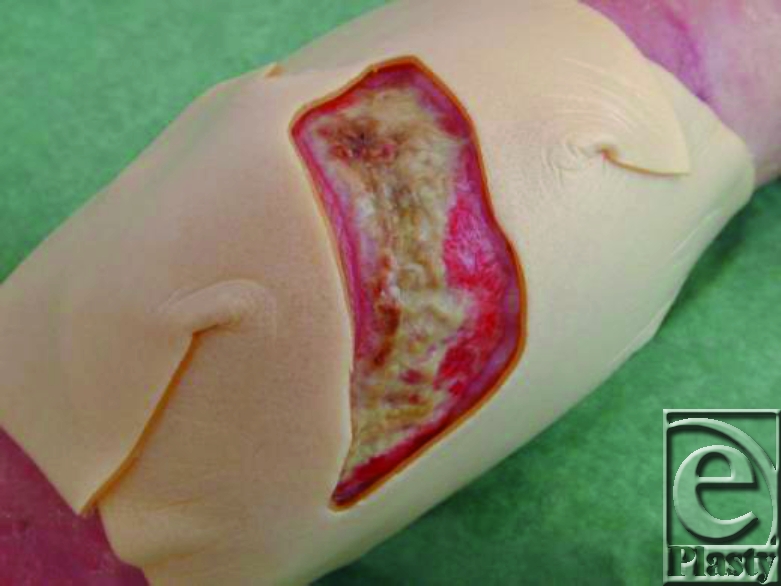
Film template cut out of hydrocolloid.

**Figure 5:**
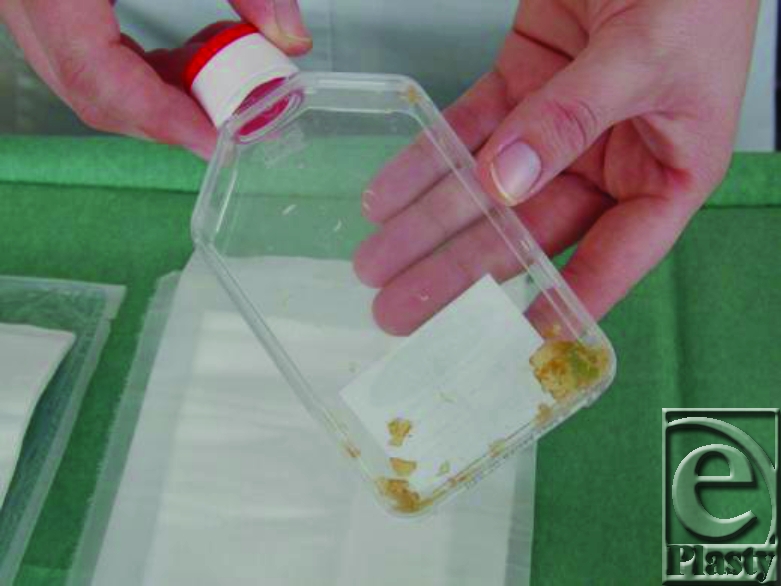
Maggots in container.

**Figure 6:**
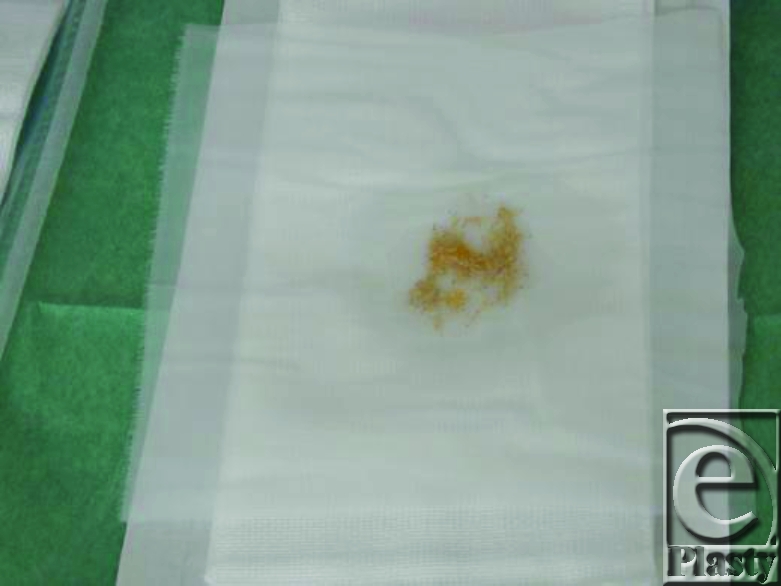
Maggots on the net.

**Figure 7:**
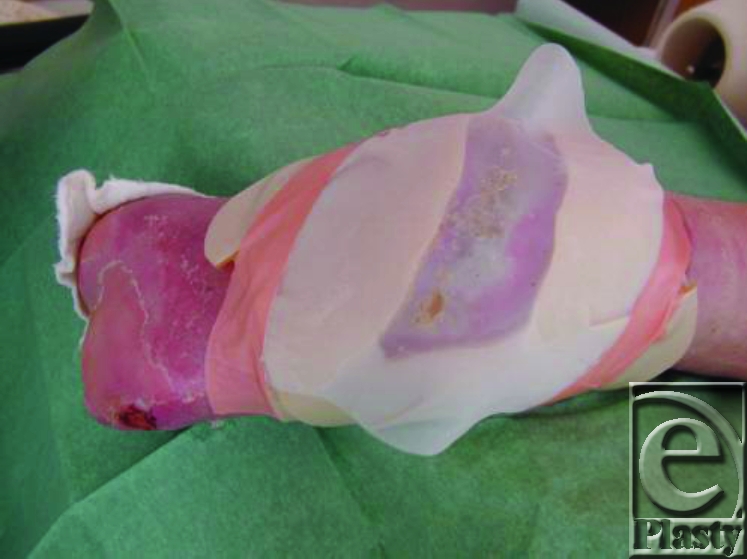
Net with maggots placed in the wound.

**Figure 8:**
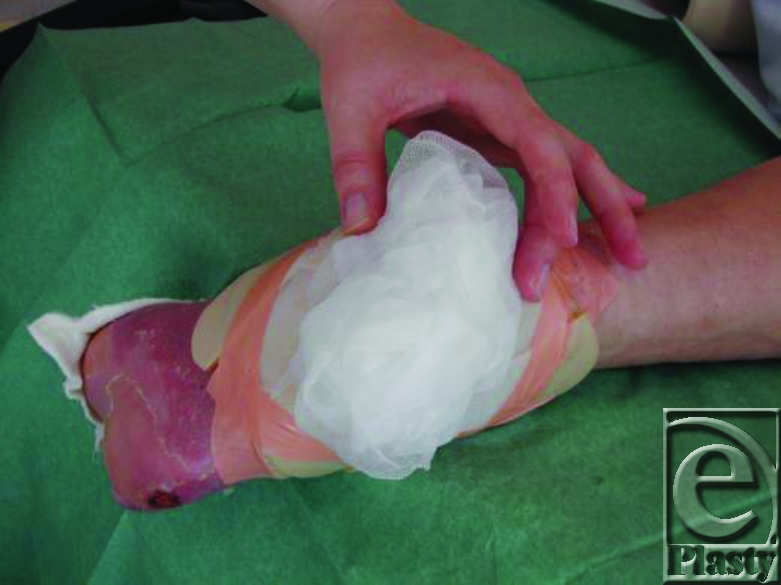
Nonwoven gauze place on the net.

**Figure 9:**
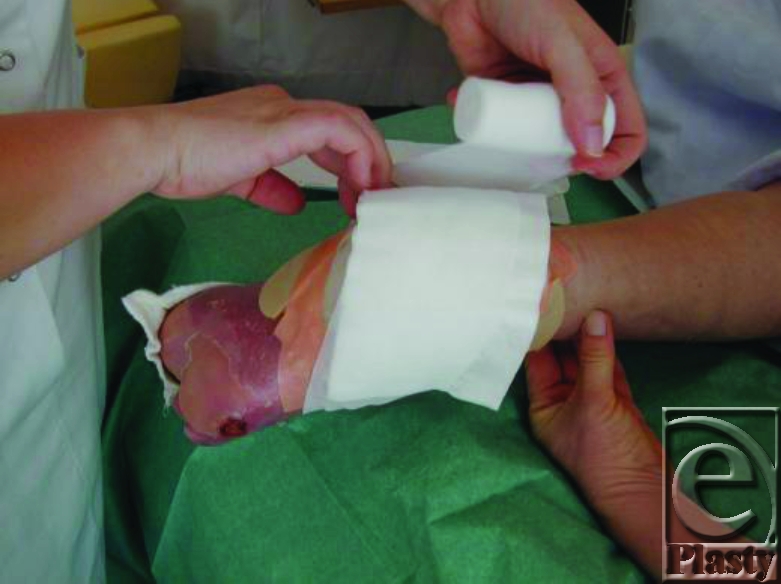
Fixation by gauze bandage.

**Table 1:** Maggot debridement: expected mode of action

**Debridement**	Ingesting necrotic tissue. High proteolytic effect liquefies necrotic tissue.
**Kill Bacteria**	Ingested bacteria are killed in the gut of the maggots
**Environmental**	Production of ammonia increases pH and inhibits growths of bacteria
	Stimulate wound healing combined effect of mentioned earlier effects. Direct stimulatory mechanical effect?
**Inhibit Biofilm**	Inhibit and break down biofilms of various bacteria

**Table 2:** Recommendations for the procedure of maggot debridement therapy

*Application of the maggots*
Freely crawling maggots:
1. Primarily the wound boarder is drawn on a film (Fig 3) and the template is used for the wound “opening” in a hydrocolloid dressing. The skin wound surroundings are protected by a barrier film (eg, Cavelon No Sting).
2. The opening in the hydrocolloid is placed exactly on the wound edges to protect the wound surroundings (Fig 4). In case of very superficial wounds, 2 layers of hydrocolloid can be put on to make the cavity large enough for the maggots.
3. The maggots are removed from the container by using of few mL of saline (Fig 5). The maggots is placed on the net which is included in the maggots shipment should use 10 maggots/cm^2^ surface area of the wound (Fig 6).
4. The net is turned to place the maggots into the wound cavity (Fig 7). The net is fixed with water safe wound tape on the hydrocolloid surface. Be sure it is tight.
5. A nonwoven gauze slightly moisture by saline is then placed on the surface of the net (Fig 8) to give humidity to the maggots, which is needed to survive in the initial phase. Later the maggots produce the humidity by their own secretion.
6. Then, more (dry) nonwoven gauze is placed on the top of the moistured gauze and fixed by gauze bandage (Fig 9). The patient is then mobilized without pressure on the maggots. Compression bandages is not allowed.
Maggots in a bag:
1. The bag is placed directly in the wound cavity.
2. Similar to the procedure point 5 and 6 for the freely crawling maggots.
*Observation of the maggots:*
At day 0, day 1 and 2.
The most superficial dressing is lifted and removed if fully soaked (otherwise the maggots will drown). The activity of the maggots is observed.
If very wet conditions in the wound dry nonwoven gauze is placed on the net, if dry in the wound use moisture nonwoven gauze. If secretion is observed at the surface of the bandage, it should be changed at once.
Pain is normally not a problem, but in case of vasculitis wounds there can be intensive pain reactions (use VAS-scale to observe).
In case or bleeding the treatment often has to be stopped.
*Removal of the maggots:*
The wound is flushed by saline/water and the maggots are removed into a plastic bag. A forceps is used to remove eventually maggots left. The maggots are destroyed together with other hospital garbage.
Bio bags is removed in Toto and destroyed together with other hospital garbage.
The wound is observed for an eventual new treatment by maggots.
An observation record of the maggot treatment should be filled out for each patient.
*Video*
The procedures described earlier are demonstrated on the attached video.[Click here]
